# Impact of Microplastics on Pregnancy and Fetal Development: A Systematic Review

**DOI:** 10.7759/cureus.60712

**Published:** 2024-05-20

**Authors:** Raj Kishor Sharma, Usha Kumari, Sudhir Kumar

**Affiliations:** 1 Microbiology, Patna Medical College, Patna, IND; 2 Biochemistry, Indira Gandhi Institute of Medical Sciences, Patna, IND; 3 Electrical Engineering, Indian Institute of Technology, Patna, Patna, IND

**Keywords:** reproductive health, endocrine disruptors, fetal development, pregnancy, microplastics

## Abstract

Microplastic (MP) pollution is a growing global concern because of its potential to impair human health, particularly with regard to fetal development. However, the origins of prenatal MP exposure and its effects on fetal development have not been well studied. This study aimed to provide a systematic review of the literature regarding the impact of microplastics on pregnancy and fetal development.

PubMed, Embase, ScienceDirect, Web of Science, Scopus, and Google Scholar were searched from 2010 until March 2024. Original publications exploring the impact of microplastics on pregnancy and fetal development were included in the study. After selecting papers, two independent reviewers extracted data regarding study characteristics, microplastics identified, and reproductive impacts. The quality of studies was assessed using the Critical Appraisal Checklists for Studies created by the Joanna Briggs Institute (JBI). Twelve studies, including 234 subjects, were selected from a total of 2,809 citations for the final qualitative analysis. Articles were published between 2021 and 2024, and most were conducted in China. The results of the included studies confirmed the existence of microplastics with varying sizes (2.1 to 100 micrometers) in the placenta and the fetal body. Studies revealed correlations between lifestyle choices and the presence of microplastics in the placenta. They also reported correlations between the level of microplastics and diminished microbiome diversity, reduced birthweights, affected gestational age, and fetal growth and development. Microplastics may be detrimental to a developing fetus during pregnancy. Nonetheless, more thorough research is required to comprehend the impact of microplastic exposure on pregnancy and fetal development.

## Introduction and background

Global health concerns about plastic pollution are growing, especially with regard to the increasing number of microscopic plastic particles known as microplastics (MPs) [1]. The amount of plastic produced worldwide has increased significantly with industrialization, from 1.5 million tons in 1950 to around 390 million tons in 2021 [2]. Although the use of plastics has greatly increased convenience, it has also led to the environmental release of a significant amount of plastic waste, which has accumulated in ecosystems. Only 9% of the 460 million tons of plastic items produced annually worldwide in 2019 were recycled; by 2060, this number is predicted to rise to 1.2 billion tons [3].

The presence of MPs has gained attention in recent years due to possible health effects and human exposure. Billions of tons of plastic litter the environment as a result of excessive plastic production and usage combined with inadequate garbage disposal [4-7]. The human body already contains microplastics; they have been found in the placenta, lungs, liver, urine, sputum, breast milk, and blood [8-15]. Studies investigating the possible harmful effects of microplastics on reproduction at the cellular level and in animal models have increased recently. The first reports of microplastics found in human placenta and meconium were reported in 2021 [12,16-19]. Chronic MP particle ingestion in mice can cause metabolic disorders, intestinal barrier failure, and dysbiosis of the gut microbiota [7].

Microplastic deposition in the human placenta has been reported recently, posing significant issues about the biological impact of these pollutants on the health of expectant mothers and their children [4]. Three routes exist for microplastics and nanoplastics to reach the mother's body: ingestion, skin contact, and inhalation. These particles have the ability to enter the circulation and travel to many organs in the body, including the placenta during pregnancy, via the circulatory system. Microplastics and additives enter the fetal body and amniotic fluid after crossing the placenta. Based on research on animals and in vitro cultures, there is growing proof that plastic particles are hazardous to fetuses and the placenta. According to studies, maternal exposure to microplastics during breastfeeding and pregnancy altered the neural cell compositions and the histology of the offspring's brains [16,18,20,21]. Studies have shown that exposure to MPs during pregnancy and the first few months of life may result in permanent alterations to the reproductive axis and central nervous system in the offspring of different species [16,21,22].

A lack of evidence has prompted scientists and regulatory bodies to express concerns over the presence of MPs in food, possible human consumption, and potential health effects. There are worries about the health of humans due to continuous exposure to plastic particles, especially concerning the consequences for childbirth. Testing whether the toxicity of micro- and nanoplastics observed in cell culture and animal research translates to unfavorable results for fertility, pregnancy, and fetal development in human populations is crucial from a clinical and public health standpoint. Preterm delivery, stillbirth, fetal growth restriction, spontaneous abortion, and pre-eclampsia are just a few of the pregnancy issues that can result from immune system imbalance.

It has been discovered that human tissues, such as the placenta and embryonic meconium, contain microplastics that are generated when ambient plastic pollution breaks down. A recent systematic review reported that plastic particles might move across the placenta [21]. It was suggested that more research be done on the translocation of various plastic particle types and heterogeneous combinations across the placenta, exposure at various stages of gestation, and connections with poor birth outcomes and other developmental outcomes. These particles may be harmful to reproduction, as evidenced by research on cell cultures and animals; however, it is unknown if these findings are linked to diminished reproductive health or adverse pregnancy outcomes. The present study was conducted to ascertain the effect of microplastics on pregnancy, fetal development, and fetal outcome.

## Review

Methods

Preferred Reporting Items for Systematic Review and Meta-Analyses (PRISMA) checklist guidelines were followed [23].

Search Strategy

A comprehensive literature search was carried out in five different databases (PubMed, Embase, ScienceDirect, Web of Science, and Scopus) and Google Scholar from 2010 to March 2024. During the literature search, restrictions were not placed on the country, time, or language of publication. Editorial letters, conference proceedings, and practice guidelines were excluded.

The following key terms were used to identify relevant studies: (microplastics) AND (pregnancy OR fetal growth OR fetal outcome) and only research articles were retrieved and reviewed. All possible combinations of keywords were utilized. Search strings were prepared using these keywords and used for database search (Table 1).

**Table 1:** Details of the databases and the search strings used.

Databases searched	Keywords used
PubMed (https://pubmed.ncbi.nlm.nih.gov)	(“microplastics") AND ("pregnancy" OR "fetal growth" OR "fetal outcome")
Embase (https://www.embase.com)	(“microplastics") AND ("pregnancy" OR "fetal growth" OR "fetal outcome")
Science Direct (https://www.sciencedirect.com/search)	(“microplastics") AND ("pregnancy" OR "fetal growth" OR "fetal outcome")
Web of Science (http://apps.webofknowledge.com/WOS)	(“microplastics") AND ("pregnancy" OR "fetal growth" OR "fetal outcome")
Scopus (https://www.elsevier.com/en-in/products/scopus/search)	(“microplastics") AND ("pregnancy" OR "fetal growth" OR "fetal outcome")
Google Scholar (https://scholar.google.com/)	(“microplastics") AND ("pregnancy" OR "fetal growth" OR "fetal outcome")

Population, Exposure, Outcome, and Study Design (PEOS) Strategy

The PEOS strategy involves studying human subjects (P) to examine the environmental exposure to microplastics (E) and its impact on clinical outcomes related to pregnancy, fetal growth, and fetal outcomes (O). The research utilizes an observational study design, including both prospective and retrospective cohorts, as well as cross-sectional studies (S).

Study Selection

After removing duplicates, titles and abstracts were screened as per eligibility criteria. The full-text articles of all the identified abstracts were reviewed independently.

Criteria for Considering Studies

The inclusion criteria included all published studies, irrespective of study design, reporting on the impact of microplastics on pregnancy, fetal growth, and fetal outcome.

The exclusion criteria included (1) studies not related and not providing sufficient data; (2) studies without results; (3) studies in languages other than English; (4) studies with duplicate data; and (5) case reports, commentaries, guidelines, editorials, book chapters, letters to the editor, reviews, and meta-analyses.

The reference lists of previous systematic reviews and meta-analyses were also screened for relevant studies. The inclusion of both published and unpublished studies, as well as gray literature, was also considered.

Data Extraction and Study Quality Assessment

Data on the first author, publication year, country, participant characteristics (age, number of fetuses, mode of birth), outcomes, and other related details were extracted.

The quality of all selected studies was assessed using the Joanna Briggs Institute's (JBI) Critical Appraisal Checklists for Studies [24]. The risk of bias in a study was considered high if the “yes” score was 49% or lower. Studies with a score between 50 and 69% were considered at moderate risk, and those with a score of 70% or higher were considered at low risk of bias. All the studies included were evaluated for the risk of bias and then classified accordingly, i.e., studies with low risk, high risk of bias, and studies with some concerns. Disagreements between the two independent reviewers were addressed by discussion and consensus.

Results

Identification and Description of Studies

**Figure 1:**
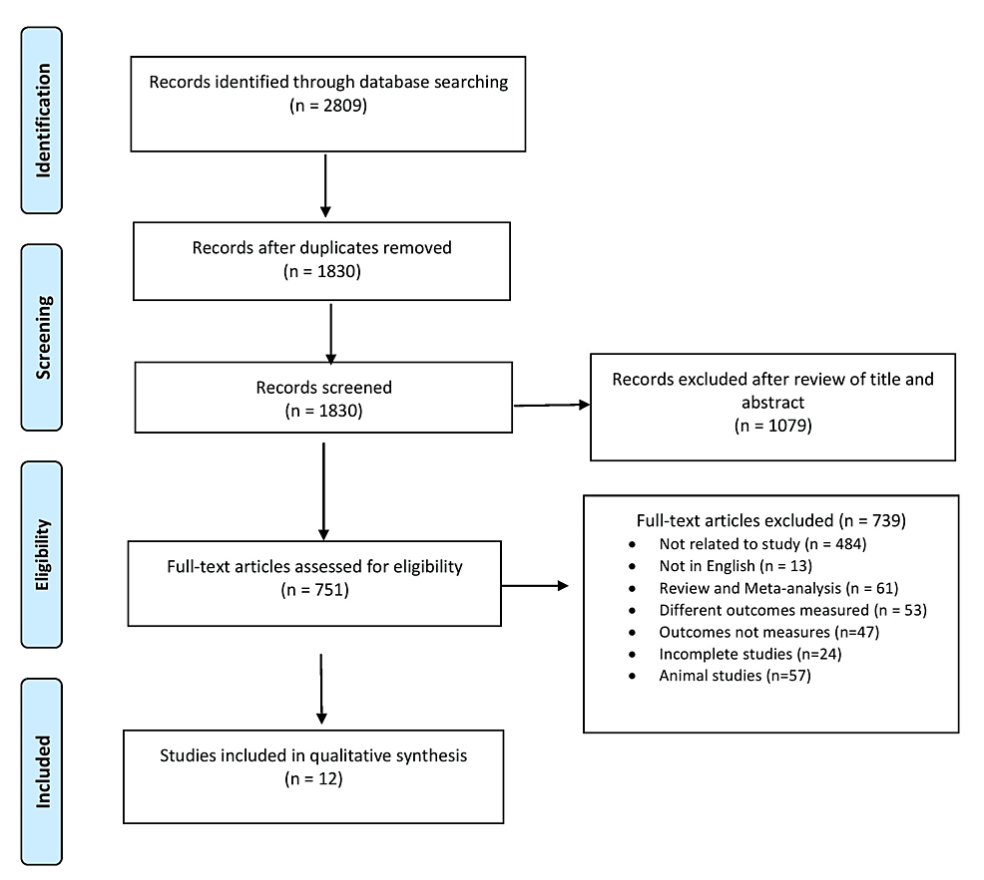
PRISMA flow chart depicting the process of selecting or rejecting studies

A total of 2,809 citations were identified, of which 979 duplicate studies were eliminated. These included 648 from PubMed, 710 from Embase, 816 from Google Scholar, 216 from Scopus, 233 from ScienceDirect, and 186 from Web of Science. After evaluating the titles and abstracts of 1,830 articles, a total of 1,079 studies were excluded. The remaining 751 articles met the requirements for the full-text review. Following the application of exclusion criteria, 739 full texts were eliminated, leaving 12 articles for the final qualitative analysis. The flow diagram (Figure 1) shows how the study selection procedure was carried out.

This review, which includes 12 studies, aimed to enumerate the impact of microplastics on pregnancy, fetal growth, and fetal outcome. The publishing years ranged from 2021 through 2024. A total of 234 subjects (sample sizes in individual studies ranged from 2 to 43) were included, with most studies conducted in China (n=4). The remaining studies were from Italy (n=2), and one each from the USA, Germany, Czech Republic, Malaysia, Canada, and Iran.

Of the 12 studies, six had a cross-sectional design, four had a prospective cohort design, one had a retrospective cohort design, and one had a case-control design. Three studies investigated mother-infant pairs, whereas nine studies solely included pregnant women as participants. Two studies included individuals regardless of birth mode, one study included individuals who underwent cesarean sections, while the majority of studies exclusively included vaginal deliveries.

Six studies looked only at placental samples for microplastics; three looked at placental and meconium samples; one looked at amniotic fluid only; one looked at the placenta and amniotic fluid; and the final one looked at pregnant women's stool samples. Microplastics made of various polymer types (such as polyethylene, polyurethane, and polyamide) were found in placental, meconium, amniotic, and stool samples in all of the included investigations. The microplastic fragments found in the investigations ranged in size from 2.1 to 100 micrometers. Four studies used laser direct infrared spectroscopy to determine placental microplastic levels; three studies used Fourier-transform infrared microspectroscopy; and one study used variable pressure scanning electron microscopy and transmission electron microscopy. The included research established an association between the physiological or clinical correlates of microplastics in clinical samples, such as placental and meconium microbiota, alterations in cell structure, and outcomes related to birth. The extracted data of the included studies are summarized in Table 2.

**Table 2:** Characteristics of included studies. MPs: microplastics.

Reference	Year	Country	Participants (n)	Study design	Mode of delivery	Clinical samples	Detection method	Microplastics	Size of MPs	MPs detected	Risk of bias
Xue et al. [25]	2024	China	40	Prospective cohort	Vaginal	Amniotic fluid	Laser direct infrared spectroscopy	Chlorinated Polyethylene Polyethylene	20-100 μm	32 out of 40 amniotic fluid samples	Low
Hasanah et al. [20]	2024	Malaysia	30	Prospective cohort	NR	Stool	Fourier-transform infrared microspectroscopy	Chlorinated Polyethylene Polypropylene Terephthalate Polyamide/Nylon	0.2-4.9 mm	30 out of 30 stool samples	Low
Zurub et al. [5]	2024	Canada	10	Cross-sectional	Vaginal/ Caesarean	Placenta	Light microscopy and Raman microspectroscopy	Polyethylene Polystyrene Polyvinyl chloride	2-60 µm	10 out of 10 placentas	Low
Liu et al. [10]	2023	China	18	Prospective cohort	Vaginal	Placenta Meconium	Laser direct infrared spectroscopy	Polyethylene Polyurethane Polyamide	20-50 µm	18 out of 18 placentas and 18 out of 18 meconium specimens	Moderate
Halfar et al. [8]	2023	Czech Republic	10	Prospective cohort	Preterm birth	Placenta Amniotic fluid	Fourier-transform infrared microspectroscopy	Chlorinated Polyethylene Calcium Zinc PVC stabilizer	20-50 µm	9 out of 10 placentas and amniotic fluid specimens	Moderate
Weingrill et al. [26]	2023	USA	30	Retrospective cohort	NR	Placenta	Light microscopy and Raman microspectroscopy	Polyethylene Polyurethane Polyamide	1-44 µm	6 out of 10 placentas in 2006, 9 out of 10 placentas in 2013, and 10 out of 10 placentas in 2021	Moderate
Zhu et al. [27]	2023	China	17	Cross-sectional	Vaginal	Placenta	Laser direct infrared spectroscopy	Polyethylene Polystyrene Polyurethane	<100 µm	17 out of 17 placentas	Low
Ragusa et al. [13]	2022	Italy	10	Cross-sectional	Vaginal/ Caesarean	Placenta	Variable pressure scanning electron microscopy and transmission electron microscopy	Polyethylene	2.1-18.5 µm	10 out of 10 placentas	Low
Liu et al. [11]	2022	China	18	Cross-sectional	Vaginal	Placenta Meconium	Laser direct infrared spectroscopy	Polyamide Polyurethane	20-50 µm	18 out of 18 placentas and 12 out of 12 meconium specimens	Moderate
Amereh et al. [28]	2022	Iran	43	Case–control	Vaginal	Placenta	Light microscopy and Raman microspectroscopy	Polyethylene Polystyrene	<10 µm	In the normal group, 4 out of 30 placentas. In the IUGR group, 13 out of 13 placentas	Moderate
Ragusa et al. [12]	2021	Italy	6	Cross-sectional	Vaginal	Placenta	Light microscopy and Raman microspectroscopy	Polyethylene	5-10 µm	4 out of 6 placentas	Low
Braun et al. [17]	2021	Germany	2	Cross-sectional	Cesarean	Placenta Meconium	Fourier-transform infrared microspectroscopy	Polyethylene Polypropylene Polystyrene Polyurethane	<50 µm	2 out of 2 placentas and 2 out of 2 meconium specimens	Moderate

Impact of Microplastics in Clinical Samples on Pregnancy and Fetal Development

The identification of MPs in the placenta and the fetal body was confirmed from the findings of the included studies [4,8,10,12,17,20,25,26].

Ragusa et al. presented the first study addressing the issue of MPs in the human placenta in 2021. Six human placentas in all were analyzed in this investigation [12]. Twelve MP particles altogether, measuring between 5 and 10 µm in size, were found in four of these placentas, with polypropylene being the most often recognized type. In a different study, Ragusa et al. (2022) showed for the first time that fragments compatible with MPs are present and localized in the cellular compartment of the human placenta [13]. They also speculated that there may be a relationship between their presence and significant ultrastructural changes in certain intracytoplasmic organelles (endoplasmic reticulum and mitochondria). These changes in typically healthy-term pregnancies have never been documented previously. Inspired by these findings, Liu et al. examined human placentas as well as baby food, breast milk, meconium, and newborn feces for the presence of MPs [10]. Eighteen infants and mothers were included in the research. The findings showed that MPs were present in every sample that was gathered, with polyamide and polyurethane being the most common materials [10,11].

Weingrill et al. showed that during a 15-year period, there was a rise in the quantity of MP-containing placentas, which was correlated with both changes in the composition of plastic polymers and an increase in the number of MP particles per volume of the placenta [26]. Six out of ten placentas (60%) in 2006, nine out of ten placentas (90%) in 2013, and ten out of ten placentas (100%) in 2021 had MP particles. The first indication of the simultaneous presence of additives and MPs in both the human placenta and amniotic fluid was found by Halfar et al. MPs were detected in the placenta, amniotic fluid, or both in nine out of ten individuals [8]. Particle sizes between 10 and 50 μm were predominant for both calcium zinc PVC stabilizer and chlorinated polyethylene. Preterm rupture of the membranes affected physiological singleton pregnancies for all ten of the study's participants. In a pilot study, Braun et al. screened meconium and placental tissue taken during two cesarean sections for breech births for MPs greater than 50 µm [17]. For polyethylene, polypropylene, polystyrene, and polyurethane, screening results for human placenta and meconium were positive. MPs larger than 50 µm were seen in the placenta and meconium obtained during cesarean delivery.

Hasanah et al. looked at whether pregnant women's feces contained microplastics [20]. About 359 microplastics in all, with particle counts ranging from 4 to 21 and diameters ranging from 0.2 to 4.9 mm per 25 g of stool, were found in the subjects' stools. Groups with varying levels of seafood intake showed significantly variable amounts of microplastics. Xue et al. looked at the levels of MPs in maternal amniotic fluid and how they related to measurements of fetal development in another research [25]. They observed that 32 out of 40 samples contained MPs, the majority of which ranged in size from 20 to 100 μm. The frequency of seafood consumption (r = 0.781) and the consumption of bottled water (r = 0.386) were positively correlated with the MPs levels in the amniotic fluid. Furthermore, there was a substantial correlation between gestational age and the quantity of total MPs in the mother's amniotic fluid.

Zurub et al. described the accumulation of plastic and non-plastic particles inside the human placenta in a recent study. Following delivery by vaginal (n=5) and cesarean section (n=5), placenta tissues were taken from healthy, singleton pregnancies. Polyethylene, polypropylene, polystyrene, and polyvinyl chloride were the most prevalent MPs, with sizes ranging from 2 to 60 μm [5].

In recent research, Zurub et al. reported the build-up of plastic and non-plastic particles inside the human placenta [4,5]. Placenta tissues were extracted from healthy, singleton pregnancies after delivery by vaginal delivery (n=5) and cesarean section (n=5). The most common MPs, with diameters ranging from 2 to 60 μm, were polyethylene, polypropylene, polystyrene, and polyvinyl chloride. Liu et al. evaluated the possible sources of MP exposure during pregnancy and breastfeeding in 2023. There were sixteen different varieties of MPs, ranging in size from 20 to 50 μm, with polyamide and polyurethane being the most common. It was discovered that nursing and the use of feeding bottles and plastic toys in babies, as well as the consumption of water and the use of toothpaste or scrub cleaner, might be sources of exposure for expectant mothers [10].

Amereh et al. investigated the first evaluation of plastic particles in 43 pregnant women's fresh human placentas and their relationship to neonatal fetal development. Up to 64% of MPs in human placentas from both IUGR and normal pregnancies were smaller than 10 μm. When comparing IUGR pregnancies to normal ones, there was an adverse relationship found between MPs exposure and birth outcomes in terms of baby weight (r= -0.82), length (r = -0.56), head circumference (r = -0.50), and 1-min Apgar score (r = -0.75) [28]. Zhu et al. assessed if MPs were present and what kind of particles they were in 17 placentas. All placenta samples included MPs, with an average abundance of 2.70 ± 2.65 particles/g. The majority of these MPs varied in size from less than 100 μm [27].

Study Quality Assessment

Two reviewers independently evaluated each included study's quality. In the majority of the studies included in this analysis, there was a low (17 studies) to moderate (10 studies) risk of bias, showing a high percentage of positive answers to the questions of the JBI tool.

Discussion

People across the world come into close, daily contact with plastics and the byproducts of their disintegration, especially nano- and microplastics. The discovery of microplastics in the placenta has sparked worries that plastics might affect fetal growth during pregnancy [16,21-29]. Plastic pollution is a serious and growing worldwide issue; by 2060, the amount of plastic that leaks into the environment is expected to have doubled to 44 million tons annually from 22 million tons in 2019 [30-32]. Plastic particles can be inhaled, consumed, or come into contact with the skin by humans. There is growing evidence of several potential health risks connected with this exposure [33]. The prevalence of microplastics in human tissues, such as the lung, blood, feces, kidney, liver, and breast milk, is becoming more and more evident [9,14,16].

Carcinogenic and endocrine-disrupting compounds are among the over 13,000 chemicals used in the production of plastics, which can seep from the products at any point in their life cycle [34]. There is evidence connecting plastic additive exposure to higher cardiovascular risk, obesity, diabetes, prostate and breast cancers, miscarriages, infertility, and neurodevelopmental issues [35,36]. Research has demonstrated that MPs cause harm to reproductive systems in several animal species and significantly affect the anomalies of development and metabolism in progeny [2,8,10,11,28,37]. A worldwide health concern, female reproductive disorders may have a direct correlation to the state of the environment [16]. Furthermore, prenatal exposure to these chemicals is especially concerning for the health and development of the unborn child because pregnancy is a critical time for the development of newborn organs [37]. The processes via which MPs interfere with female reproductivity are yet unknown, though.

Overall, we have only found a small number of human pilot studies that describe microplastics in reproductively important tissues and assess their relationships to plastic exposure or to outcomes linked to fertility and pregnancy. Every study that was included had extremely small sample sizes. The inadequate data on microplastics' prevalence in the human body and placenta has left us with an imperfect understanding of their occurrence in the human body, especially in mothers and fetuses. Microplastics may harm a fetus's reproductive system during its development, which might result in infertility or other reproductive problems, according to studies, albeit the evidence is weak. The negative effects of additives are better supported by data, suggesting that micro- and nanoplastics (MNPs) might damage developing reproductive organs through these compounds [2,16,21].

Microplastics were found in stool, amniotic fluid, placental, and meconium samples in all of the included investigations. However, very little is known about the effects of early windows of susceptibility exposure to plastic particle pollution [38]. By changing the regular release of gonadotropin and reproductive hormones, MPs may impede human reproductive development. Three studies published data indicating that levels of placental microplastics may be influenced by plastic exposure during pregnancy, such as through the use of food packaging, hygiene items, eating seafood, and consuming different types and volumes of water. The growth of germ cells and the quality of embryos are indicators of the impact of microplastics on reproduction [3]. According to Liu et al., exposure to PS lowers the quality of oocytes via interfering with the maturation of oocytes and the formation of female mice follicles [10]. Furthermore, immunological diseases linked to microplastics may have a negative impact on pregnancy outcomes, according to Hu et al. [7]. According to a recent study, birthweight and the buildup of MPs in the placenta are inversely correlated. Similar relationships were seen for newborn length, head circumference, and 1-minute APGAR ratings [28]. Furthermore, Xue et al. found an inverse relationship between the amounts of MPs found in the amniotic fluid collected at delivery and gestational age, indicating that exposure to MPs throughout pregnancy may lead to an earlier onset of labor [25].

The placenta, a transitory but essential organ of pregnancy, facilitates all fetal-mother interactions necessary to maintain the growth and development of the fetus. Adult women are exposed to an average of 258 MP particles daily through eating and inhalation, according to a meta-analysis [39]. This sparked debate on whether MPs may affect human health by passing through barriers other than the gut mucosal barrier. The presence of MPs inside the human placenta and fetal meconium suggests that MPs can traverse the placenta, according to several recent studies [4,10-12,17,27,28]. Plastic particles have been shown to be capable of placental translocation in a recently completed systematic study [16,21]. Researchers have been advised to investigate the translocation of various plastic particle types and heterogeneous mixes throughout the placenta, exposure at various stages of gestation, and correlations with unfavorable delivery and other developmental consequences.

As evidenced by the presence of compounds linked to plastics that are known to affect hormone levels in both human amniotic fluid and the placenta, plastic particles may also cross the blood-placenta barrier [1,14]. The existence of MPs in the placenta raises questions about how these particles may affect placental integrity and function, including hormone synthesis and maternal-fetal exchange, and may also indicate MP translocation into the fetal enclosure. Because MPs are found in large quantities in all placenta samples, it is important to investigate the possible effects they may have on fetal development and placental function. This is an area of research that has not received much attention, especially outside of human populations. Grafmueller et al. have previously shown that polystyrene nanoparticles might get through the placental barrier [40]. Using ex vivo placenta perfusion models, the transplacental migration of nanoplastic particles (<100 nm) has been established recently, accompanied by a shift in the protein corona composition [41,42]. Furthermore, placenta in vitro co-culture models have demonstrated the cellular absorption and intracellular accumulation of nano- and microparticles [43]. The most recent article found that all five placentas delivered vaginally had MPs ranging from 5 to 10 µm [12].

Understanding the biological harm caused by exposure to certain contaminants and plastics, particularly during a time as vulnerable as pregnancy, should be the impetus for a shift in approach in this era of environmental crisis. Studies on the effects of MNP exposure during the periconception and embryonic stages are currently lacking, despite the fact that this is a very sensitive time that requires careful consideration given the increasing number of plastics in our environment. In order to provide the best environment for the well-being of pregnant women and fetuses, especially during the vulnerable time of pregnancy, it is our responsibility as researchers and clinicians to make women aware of the grave consequences of microplastic contamination and suggest personalized solutions to lessen the impact of this menace. However, the most effective way to mitigate the menace will be significantly reducing global plastic production and use and increasing the proportion of recycled plastics.

## Conclusions

Due to potential degradation of placental endocrine, metabolic, and immune systems, microplastics may have a negative impact on a growing fetus. To comprehend the impact of microplastic exposure on the growing fetus and the gestational parent, further high-quality research involving larger cohorts of pregnant women is needed.
